# State‐Programmable Conductive and Reactivatable Bio‐Adhesive for High‐Fidelity Epidermal Biointerfaces

**DOI:** 10.1002/smll.75442

**Published:** 2026-08-26

**Authors:** Salahuddin Ahmed, Marzia Momin, Jiashu Ren, Xinyi Wang, Jirong Lin, Jia Sun, Yueqi Deng, Hyunjin Lee, Xiaojun Lance Lian, Tao Zhou

**Affiliations:** ^1^ Department of Engineering Science and Mechanics The Pennsylvania State University University Park Pennsylvania USA; ^2^ Department of Mechanical Engineering The Pennsylvania State University University Park Pennsylvania USA; ^3^ Department of Biomedical Engineering The Pennsylvania State University University Park Pennsylvania USA; ^4^ Huck Institutes of Life Sciences The Pennsylvania State University University Park Pennsylvania USA; ^5^ Department of Biology Pennsylvania State University University Park Pennsylvania USA; ^6^ Center For Neural Engineering The Pennsylvania State University University Park Pennsylvania USA; ^7^ Materials Research Institute The Pennsylvania State University University Park Pennsylvania USA

**Keywords:** 3D printing, adhesion, advanced manufacturing, conducting polymer, conductive adhesive, electrode–skin interface, epidermal, packaging, reactivatable, reversible viscoelasticity, wearable

## Abstract

Owing to the inherent trade‐off between conformal wetting and mechanical stability at the electrode–skin interface, achieving both microscale conformality and mechanical stability for epidermal electrophysiology remains challenging. Ionically conductive gels provide low interfacial impedance through fluidic wetting but suffer from dehydration and instability, whereas dry soft electrodes offer mechanical stability but lack the rheological adaptability needed for intimate skin contact. Here, we report a state‐programmable graft interpenetrating network (g‐IPN) that enables a printable and reactivatable conductive adhesive (RCA). The RCA simultaneously achieves high electrical conductivity, state‐dependent adhesion, and reversible viscoelasticity. By transitioning from a mechanically robust solid film to a fluidic state via solvent activation, the RCA infiltrates complex skin surfaces and hair‐occluded regions, delivering lower interfacial impedance than traditional gels. These properties enable stable electroencephalogram (EEG) and pulse recordings across both rigid and soft electrode platforms, establishing a scalable strategy for high‐fidelity epidermal bioelectronic interfaces.

## Graphical abstract

A printable, reactivatable conductive adhesive (RCA) transitions from a robust, non‐tacky solid film into a soft, conformal biointerface upon solvent activation. By adapting to complex skin microtopography and hair‐covered scalp, the state‐programmable adhesive achieves strong adhesion and low interfacial impedance, enabling high‐fidelity EEG and pulse recording with both rigid and soft electrodes.

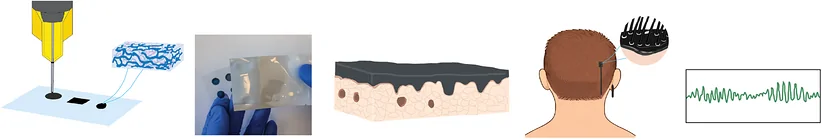

## 1 Introduction

Highly reliable epidermal electrophysiological recording is fundamental to modern clinical diagnosis [1], therapeutic monitoring [2], and human–machine interface [3]. The overall fidelity of electrophysiological systems is fundamentally governed by the interfacial conformality and electrochemical impedance of the electrode–tissue interface [4, 5, 6, 7]. To maintain high‐fidelity bio‐electrical contact over an extended period, the interface must simultaneously achieve intimate conformal contact with the complex surface of skin, mechanically adaptive integration under multiaxial dynamic deformation, and long‐term electrochemical stability without drift in interfacial impedance [8, 9, 10, 11]. Achieving these requirements within a single material state remains challenging, necessitating interfacial systems that can dynamically balance conformal contact, mechanical compliance, and long‐term electrochemical stability.

Ionically conductive gels and grease‐like pastes have long been the clinical benchmarks for electrophysiological recordings; their fluid‐like phase allows them to penetrate skin surface asperities and establish high‐quality, low‐impedance contact [12]. However, this same nonsolid nature results in poor cohesion, spreading, and weak mechanical interaction with electrodes, necessitating external fixation and leading to instability during motion [13, 14]. Furthermore, progressive drying during use stiffens the gel matrix, diminishes the effective contact area, interrupts continuous ionic pathways, and consequently elevates interfacial impedance, motion artifacts, and signal drift over time [15, 16].

To mitigate the instabilities associated with liquid electrolytes, soft and dry [17, 18, 19] electrodes have been developed to improve interfacial reliability. Dry systems remove the aqueous phase entirely and rely on solid‐state charge transport through intrinsically conductive polymers [18] or elastomeric conductive composites [20, 21]. These materials offer mechanical compliance with inherent conductivity, allowing the acquisition of electrophysiological signals under dynamic conditions [22]. While soft conductors provide a clear mechanical advantage by minimizing elastic mismatch at the interface, their performance is fundamentally constrained by their solid‐state nature. In the absence of a fluidic phase to facilitate interfacial wetting, these materials are unable to achieve true microscale conformality against the complex surface of the skin. Without rheological adaptability to infiltrate the microtopography of skin, these electrodes remain susceptible to interfacial resistance and signal fluctuations [23, 24, 25]. Such interfacial voids hinder long‐term electrochemical stability and low‐impedance contact compared to hydrated systems [26, 27]. These limitations reveal a fundamental constraint in biointerface engineering: most materials are typically kinetically confined to static phases, enforcing a trade‐off between structural integrity and interfacial conformality that hinders their simultaneous realization in practical applications.

Here, we introduce a state‐programmable graft interpenetrating network (g‐IPN) as a materials strategy to overcome longstanding constraints in epidermal interfacing. We report a printable reactivatable conductive adhesive (RCA) that can be processed as a viscous conductive gel and, after drying, forms a mechanically robust solid film. Upon solvent‐mediated activation, this solid transforms back into a soft, conformal, conductive gel capable of adapting to the microtopography of skin and hair‐occluded regions.

The g‐IPN is formed by synthesizing a polyurethane–poly(acrylic acid) (PU‐PAA) network via UV‐initiated polymerization, followed by incorporation of poly(3,4‐ethylenedioxythiophene):polystyrene sulfonate (PEDOT:PSS) to establish ionic‐electronic conduction. The resulting viscoelastic ink can be patterned into predefined geometries and subsequently dried to form solid, pre‐formed conductive films, enabling on‐demand activation at the point of use.

The RCA interface simultaneously exhibits high electrical conductivity, low skin–electrode interfacial impedance on both skin and the hairy scalp, and strong interfacial adhesion, while retaining mechanical compliance under deformation. These combined metrics enable stable conformal contact and reliable electrophysiological signal acquisition.

## 2 Main

### 2.1 RCA Design

The RCA is designed to reversibly transition between two mechanically distinct states. During processing, the material behaves as a rheologically tunable conductive gel, making it suitable for high‐resolution printing. Following solvent removal, the network becomes kinetically trapped as a mechanically robust, dry solid film that can be handled, transferred, and stored. Transient exposure to a trace amount of solvent commonly used in clinical practices initiates rapid interfacial plasticization, effectively releasing the kinetically trapped chains within the g‐IPN. This increase in segmental mobility induces structural relaxation that restores the network's viscoelastic fluidity, allowing it to permeate the rough surface of the skin [5, 28].

This transition is critical for epidermal interfacing. In the dry state, the film rests on the skin surface and cannot infiltrate pores and surface irregularities. Upon solvent activation, the softened network flows into the skin's microtopography, enabling conformal wetting and improved interfacial contact.

### 2.2 Polymer Architecture and Conductive Network Formation

The RCA is based on a polyurethane–poly(acrylic acid) (PU–PAA) g‐IPN synthesized through a UV‐assisted photoinitiated polymerization process (Figure 1a). A mixture of polyurethane, acrylic acid, ethanol, α‐ketoglutaric acid, and benzophenone is exposed to UV light. α‐Ketoglutaric acid initiates the polymerization of acrylic acid, while benzophenone generates radical sites along the polyurethane (PU) backbone, enabling covalent grafting of poly(acrylic acid) (PAA) chains onto PU. This process produces a covalently grafted interpenetrating polymer network enriched with polar functional groups capable of dynamic hydrogen‐bond interactions.

**FIGURE 1:**
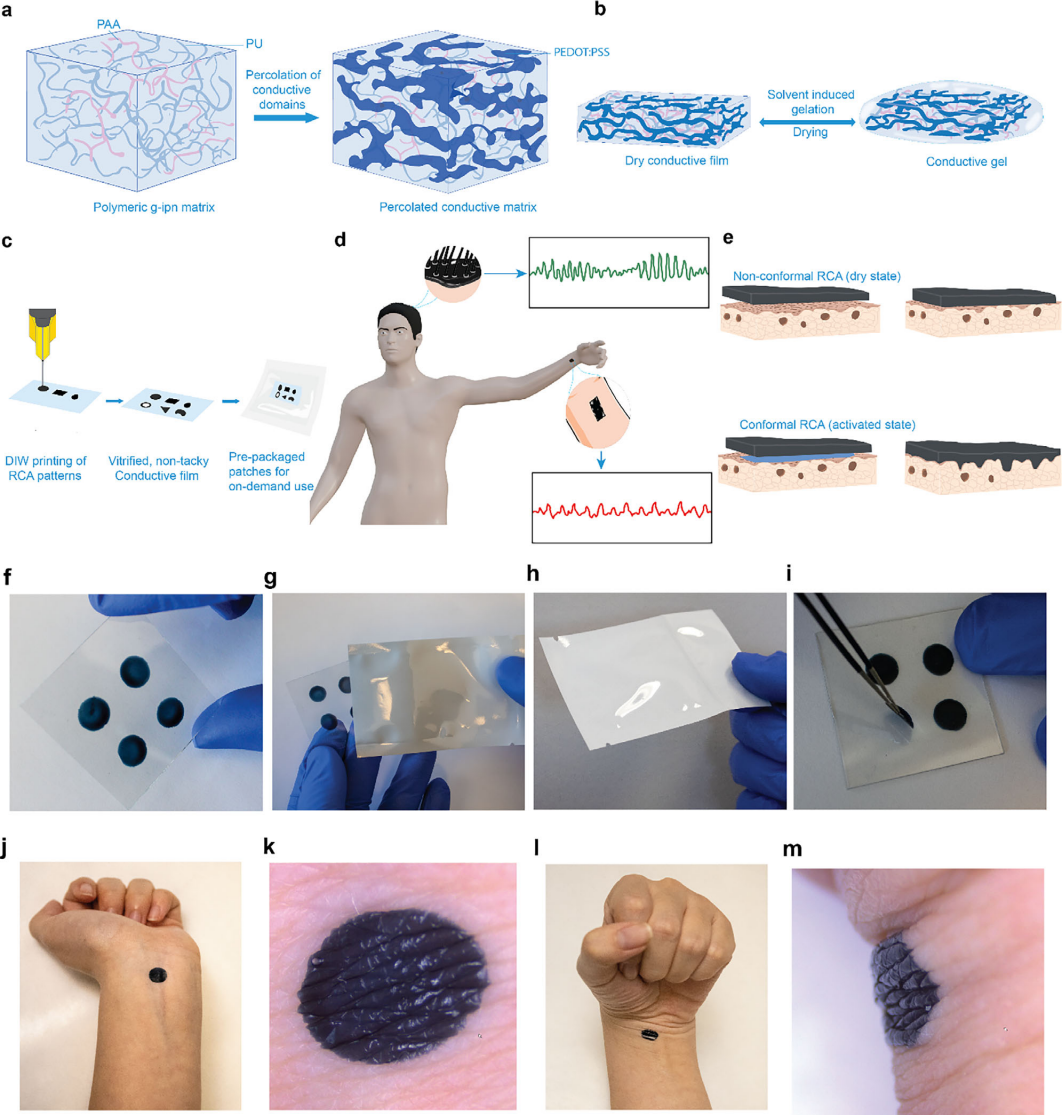
Design illustration, state transition mechanism, interfacial working principle, and packaging of the reactivatable conductive adhesive (RCA). (a) Schematic illustration of the polyurethane–poly(acrylic acid) (PU–PAA) graft interpenetrating network (g‐IPN). (b) Schematic illustration of the solvent‐triggered state transition of the RCA. (c) Illustration of fabrication and deployment of RCA. 3D printing followed by solvent evaporation forms a dry, non‐tacky conductive film that can be stored as pre‐packaged patches for on‐demand use. (d) Application modes of the RCA at the electrode–skin interface for epidermal electrophysiology. (e) Interfacial mechanism of conformal integration. In the dry state, the solid film contacts only the asperity peaks of the skin microtopography, leaving interfacial voids. After solvent activation, the softened network infiltrates pores and surface irregularities, establishing intimate microscale contact. (f–i) Packaging and handling of printed RCA films. (j) Stable adhesion during wrist stretching. (k) Magnified view showing intimate contact with skin microtopography. (l) Conformal adhesion with skin during wrist flexion. (m) Zoom‐in view showing preserved conformity under skin folding.

After purification and drying, the PU–PAA g‐IPN is redissolved in ethanol to form the matrix for the conductive adhesive formulation. To impart electrical functionality, an aqueous PEDOT:PSS dispersion containing dimethyl sulfoxide (DMSO) as a secondary dopant [29] was directly incorporated into the PU–PAA matrix precursor (Figure 1a), producing a viscoelastic conductive gel that can be patterned into predefined geometries and patch formats via high‐resolution printing (Figure 1c).

Within this material architecture, PEDOT:PSS remains physically embedded within the matrix and forms continuous conduction pathways. Because the conductive phase does not participate in network formation, the state‐transition behavior of the material is governed by the PU–PAA g‐IPN framework, while PEDOT:PSS provides electrical functionality [30]. This functional separation ensures the adhesion, rheological, and state‐programmable characteristics of the matrix are preserved at high conductive loadings.

### 2.3 Solvent‐Triggered State Transition

Following printing, solvent evaporation reduces chain mobility within the PU–PAA graft network, kinetically arresting the polymer chains and converting the deposited gel into a rigid solid film suitable for handling and transfer [31]. Brief exposure to a clinically used solvent such as isopropyl alcohol triggers hydrogen‐bond exchange within the network, temporarily weakening interchain interactions and restoring segmental mobility. This reversible plasticization transforms the solid film back into a soft, gel‐like state capable of conforming to the microtopography of the skin (Figure 1b) [5, 28]. Importantly, this transformation does not rely on swelling or dissolution as observed in conventional hydrogels, but arises from supramolecular structural reorganization of the g‐IPN that restores mechanical compliance and interfacial adaptability while preserving the covalent network structure.

The resulting state‐dependent behavior produces a bistable adhesion profile that is advantageous for practical use, facilitating reliable epidermal interfacing for electrophysiological measurements such as EEG and pulse monitoring (Figure 1d). In the dry state, the solid film only touches on the edges of the rough skin surface, leaving microscopic voids at the interface, minimizing adhesion force for easier handling and transportation. After solvent activation, the material softens and conforms to the skin's microtexture, infiltrating surface pores and establishing intimate contact across the interface (Figure 1e).

To enable practical deployment of this state‐dependent behavior, the material must also support reliable handling, storage, and transfer. For this purpose, the RCA can be printed directly onto hydrophobically treated, low‐surface‐energy polyamide release films. After drying, the solid patterns remain mechanically stable on the substrate and can be hermetically sealed, enabling ready‐to‐use packaging formats (Figure 1f–i). Without surface treatment, the gel forms strong interfacial adhesion to the high‐energy polyamide surface, preventing clean removal after drying. The low‐surface‐energy treatment ensures that the dried adhesive patterns can be easily peeled intact from the release film for subsequent applications.

As shown in Figure 1i, the dried patterns can be peeled intact from the release film and applied to the skin. Upon solvent activation, the material rapidly regains viscoelastic mobility, allowing it to conform to the skin's microtexture (Figure 1j–m). Once activated, the printed features maintain mechanical coupling during natural skin motion, such as wrist extension (Figure 1j) and flexion (Figure 1l). This behavior demonstrates that the RCA functions as a compliant interfacial layer rather than a rigid overlayer, preserving intimate contact throughout use.

### 2.4 Printable Conductive Ink Formulation

To realize a printable conductive ink while retaining the reversible state transition of the g‐IPN and conductive pathways of PEDOT:PSS, the PU–PAA matrix was blended with increasing volume fractions of PEDOT:PSS (P2:A8, P3:A7, P4:A6, and P5:A5) where P denotes the amount of aqueous dispersion of PEDOT:PSS (7.5 w/v%) and A denotes the amount of PU‐PAA matrix (See Section 4).

All formulations exhibit the shear‐thinning behavior required for direct ink printing (Figure 2a). Increasing the PEDOT:PSS concentration increases the zero‐shear viscosity, reflecting the integration of a secondary, hydrated polymeric phase within the adhesive network. The elevated low‐shear viscosity of the ink resulting from incorporation of PEDOT:PSS enables high‐fidelity pattern definition without the necessity for post‐printing chemical crosslinking.

**FIGURE 2:**
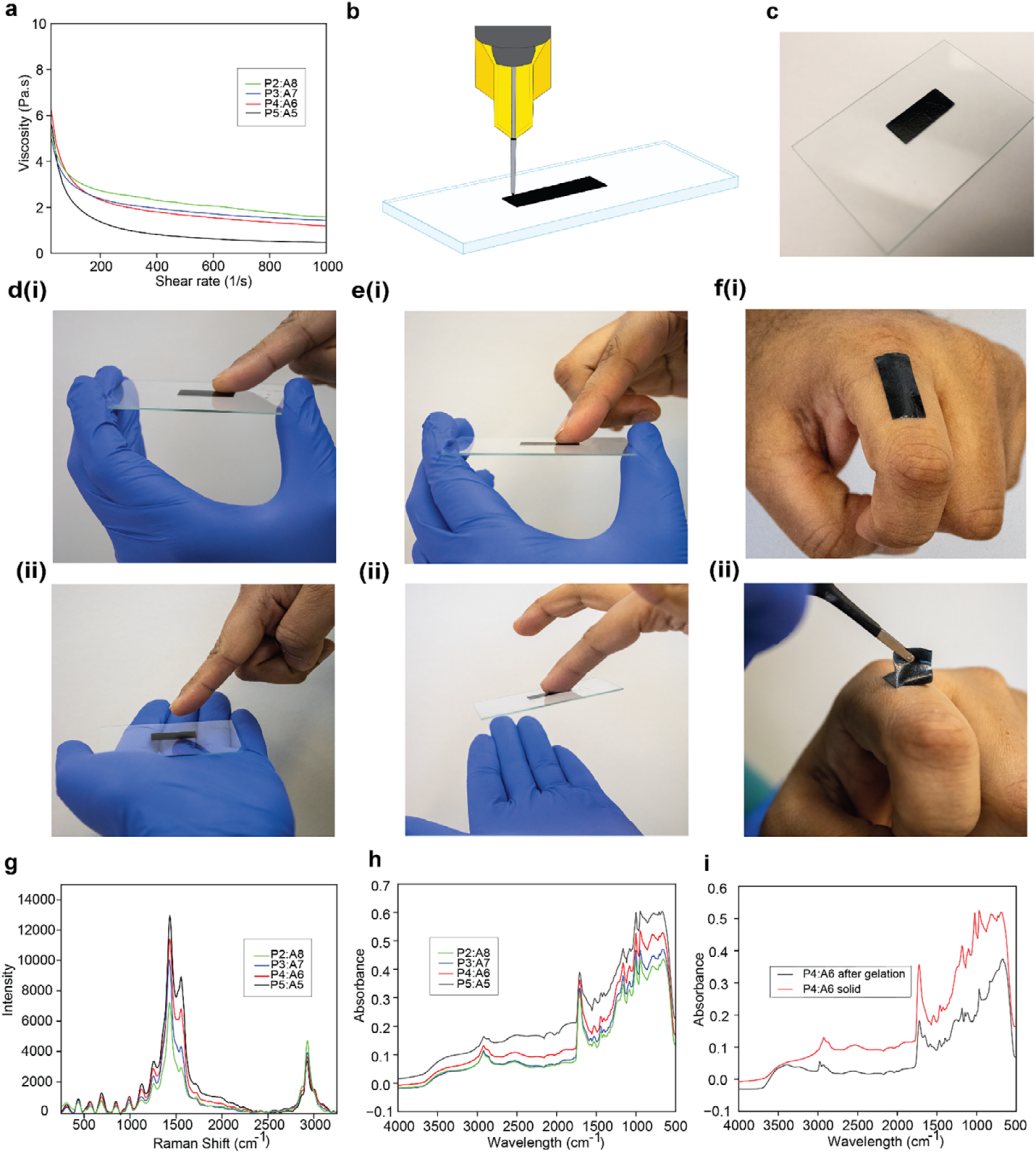
Printability, handling and characterization of the RCA. (a) Shear viscosity as a function of shear rate for RCA with varying PEDOT:PSS concentration (P2:A8, P3:A7, P4:A6, and P5:A5). (b) Schematic illustration of direct ink extrusion printing of RCA. (c) Photograph of a printed RCA strip on a glass substrate after drying. (d) Low adhesion in the dry state during handling and placement, facilitating accurate positioning prior to activation. (e) Strong adhesion in the activated state, maintaining stable mechanical contact with the skin. (f) Epidermal interfacing of RCA. (i) Upon application, the film adheres to the skin, forming stable interfacial contact. (ii) The material can be removed cleanly after use without residue, enabling repeated and convenient use. (g) Raman spectra of various RCA formulations. (h) FTIR spectra of various RCA formulations. (i) FTIR spectra of P4:A6 RCA in solid film and activated state.

The ink can be directly extruded through narrow nozzles and printed into predefined geometries on substrates, producing uniform conductive traces with well‐defined edges (Figure 2b,c). Following deposition, solvent evaporation induces vitrification of the viscoelastic ink into a mechanically stable solid state that retains printed geometry without deformation (Figure 2c). The removal of solvents during drying facilitates the formation of PEDOT‐rich crystalline domains, forming an internal conductive network within the dried film.

This transition is critical for on‐demand adhesion control during clinical use. In the dry state, the RCA exhibits minimal adhesion, enabling accurate handling and positioning (Figure 2d). Upon activation, the material forms strong interfacial adhesion, maintaining stable mechanical contact with the skin during operation (Figure 2e). This state‐dependent response enables practical deployment, allowing easy placement of the film and clean, residue‐free removal after use (Figure 2f).

### 2.5 Molecular Characterization

The molecular structure of the RCA composite was examined using Raman and Fourier transform infrared (FTIR) spectroscopy (Figure 2g–i). Raman spectra (Figure 2g) reveal the clear emergence of PEDOT:PSS vibrational modes in the conductive samples, particularly the thiophene‐ring C_α_ = C_β_ stretching bands near 1430 cm^−1,^ which increase with the increase of PEDOT:PSS concentration [32]. The spectral features of the PU–PAA matrix (Figure S1) remain present without the appearance of new peaks or shifts, indicating that PEDOT:PSS is incorporated physically within the matrix without altering the chemical structure of g‐IPN.

FTIR spectra (Figure 2h) show subtle reorganization of the hydrogen‐bonding network following incorporation of PEDOT:PSS. Compared to the PU–PAA matrix (Figure S2), the composites exhibit broadening and attenuation of the O─H stretching band (3200–3600 cm^−1^) and reduced prominence of the carbonyl stretching peak (∼1700 cm^−1^) [33]. These changes indicate redistribution of interchain hydrogen‐bond interactions within the PU–PAA g‐IPN. No new characteristic covalent bonding peaks emerge after PEDOT:PSS incorporation, confirming that the conductive phase does not participate in network formation.

Moreover, FTIR spectra (Figure 2i) elucidate the reversible modulation of hydrogen‐bond interactions that drives the solid‐to‐gel transition. Upon solvent activation, the O─H stretching band (3200–3600 cm^−1^) becomes broader and shifts toward lower wavenumbers, characteristic of strengthened and dynamically distributed hydrogen‐bonded networks [34]. Concurrently, the carbonyl stretching region (∼1700 cm^−1^) exhibits reduced peak intensity and subtle band reshaping, indicating redistribution of urethane and carboxylic acid hydrogen‐bond states. No new vibrational peak emerges, confirming that the transition does not involve chemical bond cleavage or network degradation. Instead, these reversible spectral changes support a supramolecular reorganization of the PU–PAA g‐IPN, in which solvent‐mediated hydrogen‐bond rearrangement governs the mechanical softening while preserving the covalent network integrity.

### 2.6 Electrical and Mechanical Characterization

Increasing PEDOT:PSS content results in a monotonic increase in electrical conductivity, reaching ∼3.5 S/cm at P5:A5 (Figure 3a), reflecting the increasing contribution of the conductive phase within the composite. However, the electrical response under deformation remains consistent across all compositions. Under tensile strain up to 150%, the relative resistance R/R_0_ increases gradually and in a similar manner for all compositions (Figure 3b). Similarly, under compressive loading from 0 to 20 N, R/R_0_ remains close to unity (Figure 3c). These results indicate that the conductive pathways remain mechanically integrated with the matrix during both stretching and compression, an important requirement for stable epidermal signal acquisition under motion and contact pressure.

**FIGURE 3:**
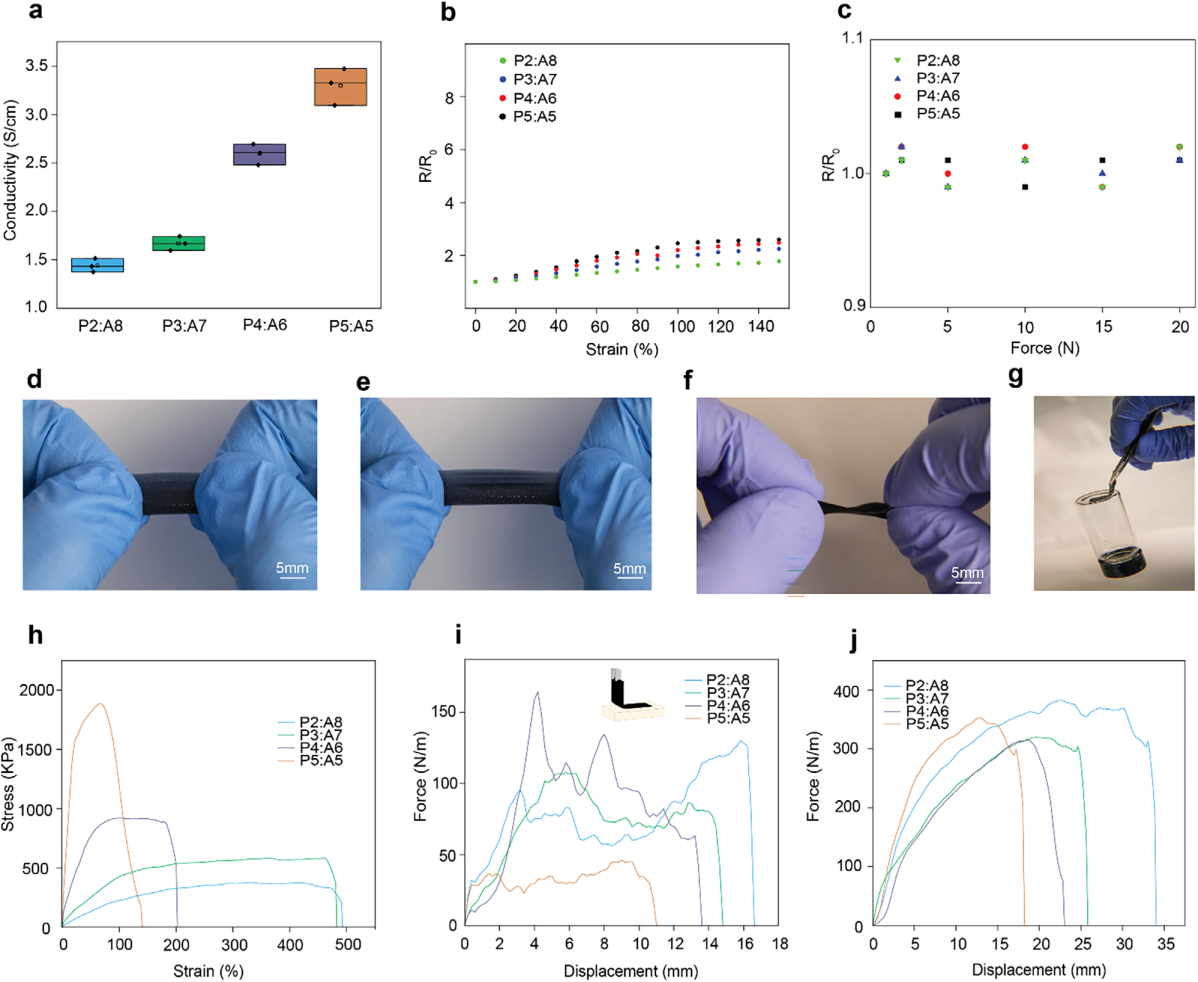
Electrical, mechanical, and interfacial performance of RCA. (a) Electrical conductivity of RCA formulations (P2:A8, P3:A7, P4:A6, and P5:A5), showing a monotonic increase in conductivity with increasing PEDOT:PSS concentration. The box represents the full data range (minimum to maximum), with the center line indicating the median and the square marker denoting the mean. Individual data points are overlaid to show experimental replicates. (b) Relative resistance change (R/R_0_) as a function of tensile strain (0%–150%). (c) Relative resistance change (R/R_0_) under compressive loading (0–20 N). (d) Photograph of a printed RCA strip. (e) Photograph of the RCA strip under tensile stress. (f) Twisting deformation of the printed RCA strip. (g) Dried RCA strip supporting the weight of a glass vial. (h) Tensile stress–strain curves of RCA composites with varying PEDOT:PSS content. (i) 90° peel adhesion force–displacement curves on skin. (j) 90° peel adhesion force–displacement curves on glass substrate.

The printed RCA exhibits excellent structural integrity, sustaining stretching and twisting without fracture or delamination (Figure 3d–f). In the dried state, a printed strip supports the weight of a glass vial (Figure 3g), highlighting the cohesive strength of the solidified g‐IPN network compared to conventional conductive gels.

Tensile stress–strain analysis reveals a mechanical trade‐off with increasing PEDOT:PSS concentration (Figure 3h). A higher amount of PEDOT:PSS increases stiffness and reduces stretchability. This compositional dependence extends to interfacial performance: increasing PEDOT:PSS content reduces the overall adhesion performance on skin or printing substrate (Figure 3i,j). The decrease in adhesion performance is attributed to partial interfacial enrichment of the conductive phase, which reduces the effective contact area and limits direct PU–PAA–substrate interactions.

We find that an optimal composition balances the trade‐off between electrical and adhesion properties, with P4:A6 emerging as the composition that maintains high electrical conductivity while preserving the stretchability and interfacial adhesion required for robust epidermal interfacing (Figure 4). We evaluated the interfacial performance of P4:A6 in practical electrophysiological configurations using both rigid Ag/AgCl EEG electrodes and soft PEDOT:PSS‐PU‐CNT (PPC) electrodes (See Supporting Information for materials and methods of fabrication) on skin and scalp (Figure 4). This comparison was performed against a commercial conductive gel to directly assess how the RCA translates into real electrode–tissue interface behavior.

**FIGURE 4:**
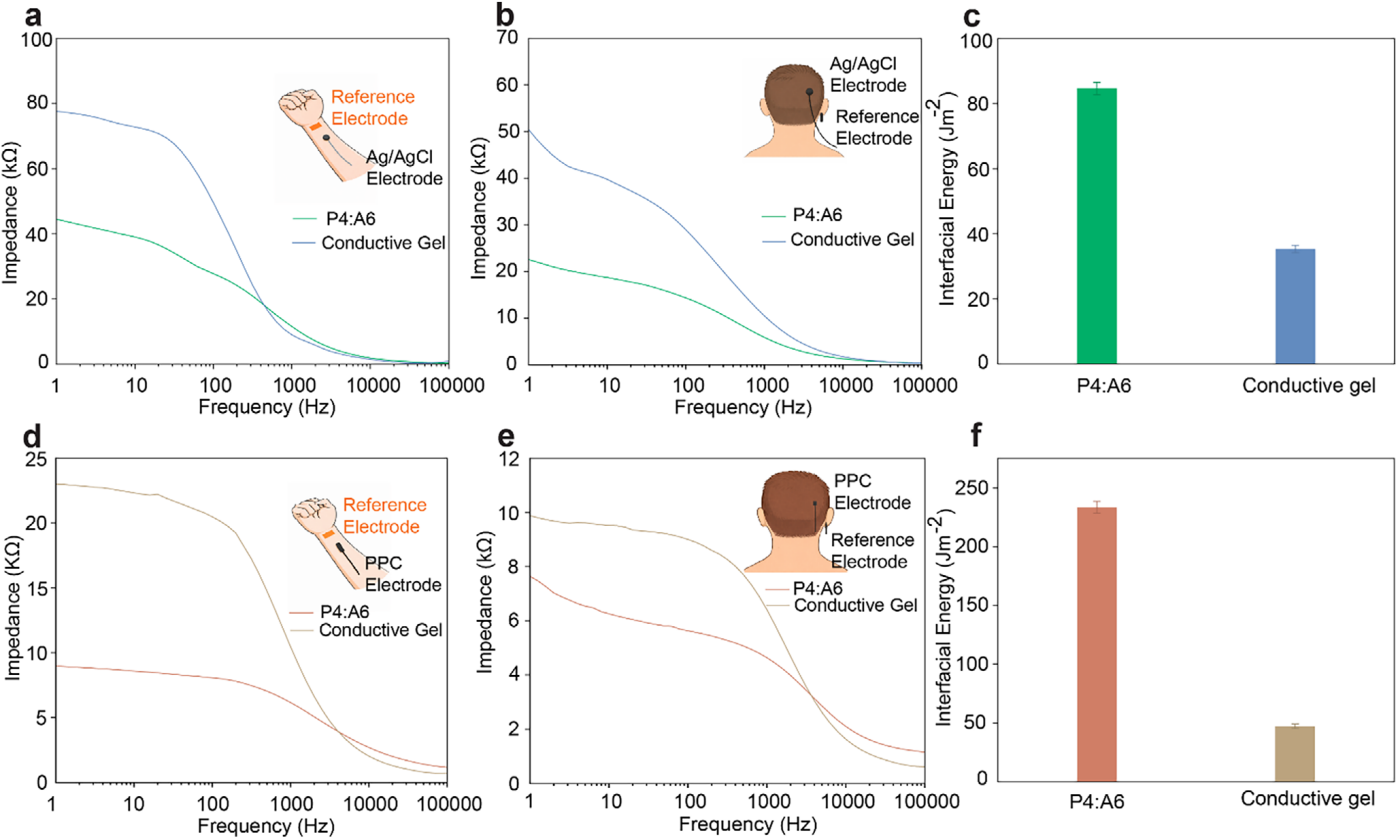
Interfacial impedance and adhesion performance of P4:A6 and commercial conductive gel. (a) Frequency‐dependent skin impedance measured on the wrist using rigid Ag/AgCl electrodes interfaced through P4:A6 and commercial conductive EEG gel. (b) Scalp impedance measured on the occipital region using Ag/AgCl electrodes interfaced through P4:A6 and conductive EEG gel. (c) Interfacial adhesion energy measured on skin for Ag/AgCl electrodes using P4:A6 and conductive gel. (Bars represent mean values, with error bars indicating the standard error of the mean) (d) Frequency‐dependent skin impedance measured on the wrist using soft PPC electrodes interfaced through P4:A6 and conductive EEG gel. (e) Frequency‐dependent scalp impedance measured using PPC electrodes interfaced through P4:A6 and conductive EEG gel. (f) Interfacial energy measured on skin for PPC electrodes using P4:A6 and conductive gel. (Bars represent mean values, with error bars indicating the standard error of the mean).

With rigid Ag/AgCl electrodes, P4:A6 consistently exhibits lower impedance than the commercial gel across the full frequency range on both skin and scalp (Figure 4a,b). The distinction is most pronounced below ∼100 Hz, the frequency regime most relevant for electrophysiological recordings. Correspondingly, interfacial energy measurements on skin show substantially higher adhesion for P4:A6 compared to the gel (Figure 4c), indicating improved conformal contact at the electrode–skin interface.

A similar trend is observed with soft PPC electrodes. When interfaced with skin and scalp, P4:A6 again produces lower impedance across frequencies compared to the commercial gel (Figure 4d,e). Interfacial energy measurements further show markedly stronger adhesion of P4:A6 to skin (Figure 4f), demonstrating that the RCA establishes a more effective mechanical and electrical junction for both rigid and soft electrode architectures.

Together, these results show that the RCA enables improved electrode–tissue interfacing compared to conventional conductive gels, particularly in the low‐frequency regime critical for electrophysiological signal acquisition.

### 2.7 Electrophysiological Recording

To validate the practical signal fidelity enabled by the RCA interface, we performed proof‐of‐concept electrophysiological recordings on the occipital region of a hairy scalp using both Ag/AgCl and PPC electrodes integrated with the adhesive layer (Figure 5). Recordings were acquired under eyes‐closed and eyes‐open paradigms to probe the device's ability to resolve canonical EEG rhythms in realistic conditions where hair and scalp texture typically hinder electrode contact.

**FIGURE 5:**
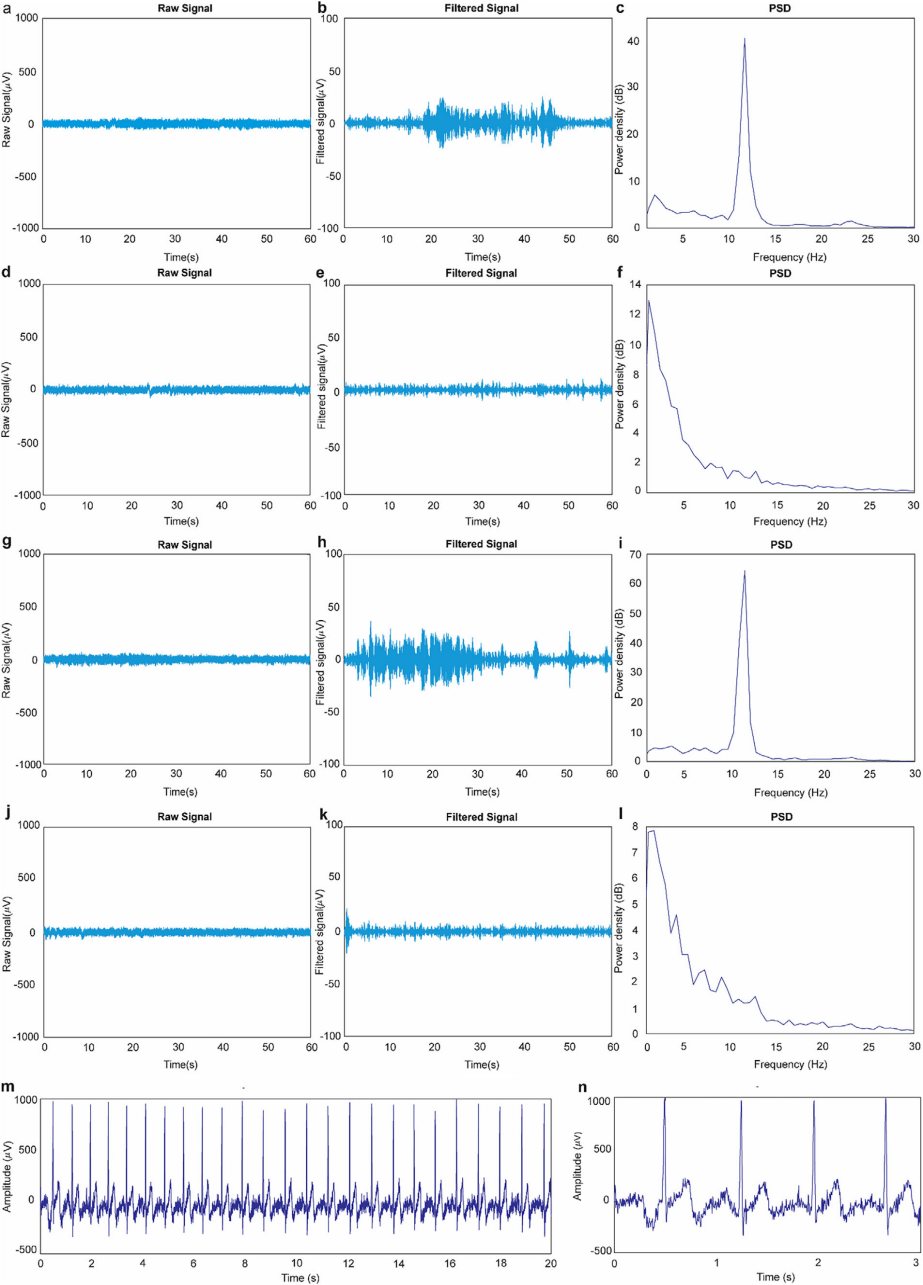
EEG and pulse signal acquisition with RCA interface. (a) EEG raw signal recorded using Ag/AgCl electrodes in an eyes‐closed paradigm using RCA. (b) EEG rhythms after band‐pass filtering in the 1–40 Hz range. (c) Power spectral density (PSD) analysis of EEG signals in (a), showing a pronounced alpha peak (∼10 Hz). (d) EEG raw signal recorded using Ag/AgCl electrodes in the eyes‐open paradigm. (e) EEG rhythms after band‐pass filtering in the 1–40 Hz range. (f) PSD of EEG signals in (d), showing suppression of alpha activity compared to the eyes‐closed condition. (g) EEG raw signal recorded using PPC electrodes integrated with RCA in the eyes‐closed paradigm. (h) EEG rhythms after band‐pass filtering in the 1–40 Hz range. (i) PSD analysis of EEG signals in (g), showing a clear alpha peak (∼10 Hz). (j) EEG raw signal recorded using PPC electrodes integrated with RCA in the eyes‐open paradigm. (k) EEG rhythms from (j) after band‐pass filtering in the 1–40 Hz range. (l) PSD analysis of EEG signals in (j), demonstrating attenuation of alpha activity during visual engagement. (m) Pulse waveform recorded using RCA‐integrated electrodes over the radial artery. (n) Zoomed‐in view of pulse waveform demonstrating clear periodic peaks and high temporal resolution.

With Ag/AgCl electrodes interfaced through the RCA, the raw and filtered signals recorded during the eyes‐closed state show clear rhythmic activity (Figure 5a,b). Power spectral density (PSD) analysis reveals a pronounced peak near ∼10 Hz (Figure 5c), corresponding to the alpha rhythm characteristic of relaxed wakefulness. When the eyes are opened, this spectral feature is strongly suppressed (Figure 5d–f), consistent with the well‐established attenuation of alpha activity during visual engagement. The ability to clearly resolve this transition demonstrates that the RCA maintains a stable, low‐impedance interface capable of capturing microvolt‐scale neural oscillations.

A similar trend is observed with soft PPC electrodes integrated with the adhesive. During the eyes‐closed state, the recordings again exhibit strong rhythmic activity with a distinct alpha peak in the PSD (Figure 5g–i). Upon opening the eyes, this feature diminishes, and the spectrum flattens (Figure 5j–l), confirming that the interface supports reliable EEG acquisition across both rigid and soft electrode architectures.

For comparison, EEG recordings obtained using the same electrodes with a conventional conductive gel (Figure S7) exhibit visibly lower signal clarity and a diminished alpha peak under identical conditions. The gel‐based interface shows higher baseline noise and reduced spectral definition.

Beyond neural signals, the RCA interface also enables acquisition of higher‐amplitude physiological waveforms. As a representative example, pulse signals recorded using the same adhesive–electrode configuration show periodic, high‐fidelity waveforms with excellent temporal definition (Figure 5m,n), highlighting the versatility of the interface for multimodal biosignal monitoring.

### 2.8 In Vitro Biocompatibility Test

The cytotoxicity of P4:A6 RCA samples was evaluated using human dermal fibroblasts (HDFs). RCA samples were incubated in Dulbecco's Modified Eagle Medium (DMEM) supplemented with 10% fetal bovine serum (FBS) and 1% penicillin–streptomycin (P/S) for 24 h to obtain the extracts (Figure S8a). HDFs were cultured in DMEM supplemented with 10% FBS and 1% P/S and exposed to varying concentrations of RCA extract (100%, 50%, 25%, and 12.5%). Cells cultured without RCA extract served as the control. After 24 h of exposure, cell viability was evaluated using a CCK‐8 assay.

According to ISO 10993–5:2009 guidelines, material is considered non‐cytotoxic when relative cell viability remains above 70% [35]. Across all extract concentrations, HDF viability ranged from 96% to 109% relative to the untreated control, demonstrating that the RCA extracts exhibited no detectable cytotoxicity toward HDFs under the tested conditions (Figure S8b,c).

## 3 Discussion

The RCA resolves the longstanding trade‐off between conformal wetting and solid‐state stability at the skin–electrode interface. Conventional conductive gels can provide low interfacial impedance, but they usually suffer from dehydration and poor mechanical stability. On the contrary, dry electrodes can offer structural robustness, but they lack the ability to achieve microscale conformal contact with skin. Through a reversible solvent‐triggered transition between solid and viscoelastic states, the RCA combines the advantages of both systems. The state‐programmable behavior of RCA enables fabrication and storage in a stable solid form while allowing rapid transformation into a compliant interfacial state during application. Furthermore, in vitro cytotoxicity evaluation using human dermal fibroblasts (HDFs) demonstrated cell viability ranging from 96% to 109% across RCA extract concentrations of 12.5% to 100%, indicating no detectable cytotoxicity under the tested conditions and supporting the potential of RCA for skin‐interfacing bioelectronic applications. The resulting interface enables intimate contact with the outermost skin layer that enables strong adhesion and low impedance even on hair‐occluded scalp surfaces. Beyond epidermal sensing, the modular graft architecture offers a scalable strategy for tailoring bioelectronic interfaces. The compatibility of the RCA with printing‐based additive manufacturing further suggests great opportunities for high‐throughput production and pre‐packaged electronics and sensors for robust adhesive interfaces. By bridging scalable fabrication with adaptive interfacial mechanics, this materials strategy significantly expands the design space for next‐generation wearable and clinical bioelectronic systems.

## 4 Methods and Materials

### 4.1 Materials

Dimethyl sulfoxide (DMSO), ethanol, acrylic acid (AA), benzophenone, and α‐ketoglutaric acid were purchased from Sigma–Aldrich (USA). Polyurethane (PU, HydroMed D3) was obtained from AdvanSource Biomaterials (USA). Poly(3,4‐ethylenedioxythiophene):polystyrene sulfonate (PEDOT:PSS) was sourced from Agfa‐Gevaert (Belgium). All reagents were used as received without further purification. Deionized water was used for all solution preparation and washing steps.

### 4.2 Preparation of RCA

The polyurethane–poly(acrylic acid) (PU–PAA) g‐IPN was synthesized via UV‐assisted photopolymerization. A precursor solution was prepared by combining a 25 wt.% PU solution (dissolved in 95:5 ethanol/DI water) with acrylic acid, ethanol, and DI water in a volumetric ratio of 26:26:28:20, respectively. Α‐Ketoglutaric acid (0.1 w/v%) and benzophenone (1.1 w/v%) were incorporated as photoinitiators. Following homogenization via stirring at 200 rpm for 15 min, the mixture was subjected to UV irradiation for 10 h. During this process, benzophenone‐mediated radical abstraction along the PU backbone facilitated the covalent grafting of PAA chains, yielding the crosslinked g‐IPN architecture [36].

The resulting product underwent rigorous purification via dialysis in ethanol for 24 h and DI water for 24 h, with solvent replacement every 12 h to ensure the removal of unreacted species. The purified product was dried at 70°C for 72 h and subsequently reconstituted in 80% ethanol at 20 w/v% to serve as the base adhesive matrix.

To impart electrical functionality, a 7.5 w/v% PEDOT:PSS suspension was formulated by dissolving solid PEDOT:PSS in a DI water/DMSO co‐solvent system (85:15 v/v) [37]. The suspension was homogenized and passed through a 10 µm polypropylene filter to ensure a uniform conductive phase. The final RCA was prepared by systematically blending this conductive suspension with the PU–PAA matrix at defined volume ratios (Px:Ay).

### 4.3 Rheological Analysis

The rheological properties of the RCA ink were characterized using an Anton Paar rotational rheometer (Anton Paar GmbH, Graz, Austria). All measurements were performed at room temperature using a parallel‐plate geometry. The sample was loaded onto the lower plate, and excess material was carefully removed after setting the measurement gap. Viscosity was measured as a function of shear rate over a range of 1–1000 s^−1^ at 25°C to evaluate the shear‐thinning behavior of the RCA inks.

### 4.4 Spectroscopic Analysis of Molecular Structure and Interactions

The molecular structure and hydrogen‐bonding interactions of the g‐IPN and RCA composites were characterized via Fourier Transform Infrared (FTIR) spectroscopy and confocal Raman spectroscopy.

### 4.5 FTIR Spectroscopy

FTIR spectra were acquired using a Bruker VERTEX 70 spectrometer equipped with a diamond Attenuated Total Reflectance (ATR) module. Data were collected across a wavenumber range of 4000–500 cm^−1^ at a spectral resolution of 4 cm^−1^, with 32 scans averaged per spectrum to ensure a high signal‐to‐noise ratio.

### 4.6 Raman Spectroscopy

Raman spectroscopy was performed using a Horiba LabRAM HR Evolution confocal Raman microscope utilizing a 532 nm excitation laser. Raman spectra were acquired through a 50× objective lens in a backscattering configuration.

### 4.7 Electrical Characterization

For electrical characterization, RCA test strips were extrusion‐printed with dimensions of 25 mm × 10 mm × ∼0.15 mm. To minimize contact resistance and ensure measurement fidelity, electrical resistance was measured using a standard four‐point probe configuration (RIGOL Technologies). Copper wire leads were connected to the printed samples using high‐purity silver paste to establish stable ohmic contact.

The electrical conductivity (σ) was calculated using:

$$\sigma =\frac{L}{R\;W\;T}$$

where L is the length of the strip, W is the width, T is the thickness, and R is the measured resistance [38].

Electrical skin impedance was measured on both the wrist and scalp using Ag/AgCl electrodes and PPC electrodes interfaced through the RCA. Measurements were performed using a potentiostat equipped with a frequency response analyzer (Autolab PGSTAT204, Metrohm) in a two‐electrode configuration.

The distance between the two electrodes was maintained at ∼2 cm. For Ag/AgCl measurements, standard EEG electrodes (diameter ∼9 mm) were used. For PPC measurements, custom PPC electrodes of comparable size were interfaced through the adhesive. A copper plate electrode (10 mm × 10 mm) served as the counter/reference electrode in all tests. For comparison, impedance measurements using the same electrodes with a commercial EEG gel were also conducted under identical conditions. Impedance spectra were recorded with a 10 mV excitation over a frequency range of 1–100000 Hz.

### 4.8 Mechanical Characterization

Mechanical properties of the RCA were characterized using a mechanical testing system (CellScale). For tensile testing, dog‐bone specimens with a gauge length of 10 mm, width of 4 mm, and thickness of ∼0.15 mm were prepared by extrusion printing. Samples were stretched at a constant displacement rate of 1 mm s^−1^ until failure. The ultimate strain was determined from the engineering strain at rupture.

Interfacial adhesion of the RCA was quantified using a 90° peel configuration on both glass and skin substrates with a mechanical testing system (CellScale). RCA strips (≈10 mm × 1 mm × 0.15 mm) were extrusion‐printed and dried to the solid state. For testing, the samples were placed onto the target substrate and activated in situ using a small amount of isopropyl alcohol to trigger conformal adhesion.

For glass measurements, cleaned microscope glass slides were used as rigid substrates. For skin measurements, tests were conducted on the finger of a healthy volunteer after gentle cleaning with alcohol wipes and air drying. The adhered length was maintained at 5 mm, while the free end of the strip was attached to the load cell using double‐sided tape to ensure firm gripping.

Peel tests were performed at a constant displacement rate of 1 mm s^−1^ while maintaining a 90° peel angle throughout the measurement.

### 4.9 Human Test Protocols

All human tests were approved by The Pennsylvania State University Institutional Review Board protocol 00022808, and informed written consent was obtained from all subjects participating in this study.

### 4.10 Electrophysiological Recording

Electrophysiological recordings were performed on healthy subjects to evaluate the signal acquisition fidelity of the RCA interface. For EEG monitoring, the occipital region of subjects with dense hair was selected. Prior to electrode placement, the scalp was prepared with a minimal volume of the solvent trigger to initiate the g‐IPN state transition and promote conformal wetting. RCA‐integrated electrodes were positioned at the occipital site, with reference electrodes secured to the earlobe.

For pulse wave monitoring, the RCA‐integrated electrodes were applied over the radial artery. The skin surface was similarly prepared with the solvent trigger to ensure intimate conformal adhesion, with a reference electrode placed adjacent to the recording site.

Signal acquisition for both EEG and pulse recordings was performed using an Intan 1024‐channel recording controller (Intan Technologies) interfaced through custom‐designed PCBs and flexible flat cables (FFC). Signals were amplified using an Intan RHD2132 amplifier and digitized at a sampling rate of 20 kHz. Post‐processing was conducted using a 60 Hz notch filter and a Butterworth band‐pass filter (1–40 Hz for EEG; 0.5–20 Hz for pulse) to isolate physiological frequency components and suppress electrical interference.

### 4.11 Data Analysis of Electrophysiological Recording

The electrophysiological signals were analyzed using MATLAB (R2023b, MathWorks). Raw EEG recordings obtained from the Ag/AgCl and PPC electrodes were first processed using a fourth‐order Butterworth band‐pass filter in the 1–40 Hz frequency range to remove low‐frequency drift and high‐frequency noise. For analysis of alpha activity associated with the eye open and eye closed states, the filtered EEG signals were further processed using a Butterworth band‐pass filter between 8–12 Hz. Power spectral density (PSD) analysis was then performed using a custom MATLAB script based on the Welch method (pwelch function) to quantify the spectral power distribution and identify characteristic EEG rhythms under different physiological states. For pulse recordings, the raw signals were processed using a Butterworth band‐pass filter in the 0.5–20 Hz frequency range, corresponding to the physiological range of human heart rates. The filtered signals were analyzed to extract the periodic pulse waveform.

### 4.12 In Vitro Biocompatibility Test

The in vitro biocompatibility of the P4:A6 RCA was evaluated using human dermal fibroblasts (HDFs). HDFs were maintained in Dulbecco's Modified Eagle's Medium (DMEM) supplemented with 10% fetal bovine serum (FBS) in an incubator at 37°C and 5% CO_2_ [39].

The cytotoxicity of RCA was assessed by cell counting kit‐8 (CCK‐8, Glpbio). The RCA was printed as a 1.9 cm^2^ circle with a height of 150 µm and transferred into a 12‐well plate, and sterilized under UV light. To obtain the extract, the sample was further incubated with DMEM supplemented with 10% FBS and 1% penicillin–streptomycin (P/S) (at a total volume of 500 µL per well in a 12‐well plate) for 24 h. The extract was then filtered through a 0.22 µm filter (considered as 100% RCA extract). HDFs were plated on a 96‐well plate at a seeding density of 5000 cells per well. 24 h post‐seeding, the cell culture media were replaced with 100 µL of either the control media (DMEM+10% FBS + 1% P/S) or varying concentrations (100%, 50%, 25%, 12.5%) of the sample extract. After a 24‐h incubation, 10 µL of CCK‐8 solution was added to each well. Following 3 h of incubation (37°C and 5% CO_2_), the plate was shaken gently on the orbital shaker for 1 min to ensure homogeneous distribution of color. Absorbance was measured at 450 nm using the microplate reader (Infinite M Plex) [16].

Cell viability was calculated using Equation:

$$Cell\;Viability\;\left(\%\right)=\left[\left(At-Ab\right)\;/\;\left(Ac-Ab\right)\right]\;\times \;100$$

Where At = absorbance of the treatment well, Ab = absorbance of the blank well (media only, without cells), and Ac = absorbance of the control well. The values were reported as the average of three or four replicates.

## Author Contributions

S.A. and T.Z. conceived the project. S.A. and T.Z. developed the material. S.A. and M.M. made the design for 3D printing. S.A. conducted 3D printing and conducted electrical characterization. S.A. conducted mechanical characterization, FTIR, Raman, and rheological experiments. S.A., M.M., J.R., X.W., J.S, Y.D., and H.L. conducted electrophysiological experiments. S.A, J.R., and M.M. did the data analysis of electrophysiological recordings. J.L and X.L.L conducted the biocompatibility tests. S.A. made the figures for the manuscript with input from all authors. S.A. wrote the original draft. T.Z and S.A. revised the manuscript with input from all authors.

## Conflicts of Interest

The authors declare no conflicts of interest.

## Supporting information




**Supporting File**: smll75442‐sup‐0001‐SuppMat.docx.

## Data Availability

All data are available in the main text or the supplementary materials. Additional raw data is available from the corresponding author upon reasonable request.
