# Supplementary material for: State‐Programmable Conductive and Reactivatable Bio‐Adhesive for High‐Fidelity Epidermal Biointerfaces
Source: Small. 2026 Aug 26;22(61):e75442. doi: 10.1002/smll.75442 (PMC13725413; doi:10.1002/smll.75442)
Supplement: Supplementary file 1 [file SMLL-22-e75442-s001.docx]

Supplementary Information

**State-Programmable Conductive and Reactivatable Bio-adhesive for High-Fidelity Epidermal Biointerfaces**

*Salahuddin Ahmed^1^, Marzia Momin^1^, Jiashu Ren^1^, Xinyi Wang^2^, Jirong Lin^3^, Jia Sun^1^, Yueqi Deng^1^, Hyunjin Lee^3^, Xiaojun Lance Lian^3,4,5^, Tao Zhou^1,3,4,6,7^* ^*^

^1^Department of Engineering Science and Mechanics, The Pennsylvania State University, Pennsylvania 16802, USA.

^2^Department of Mechanical Engineering, The Pennsylvania State University, Pennsylvania 16802, USA.

^3^Department of Biomedical Engineering, The Pennsylvania State University, Pennsylvania 16802, USA.

^4^Huck Institutes of Life Sciences, The Pennsylvania State University, Pennsylvania 16802, USA.

^5^ Department of Biology, Pennsylvania State University, University Park, PA 16802, USA

^6^Center for Neural Engineering, The Pennsylvania State University, Pennsylvania 16802, USA.

^7^Materials Research Institute, The Pennsylvania State University, Pennsylvania 16802, USA.

* Corresponding author. Email: [tzz5199@psu.edu](mailto:tzz5199@psu.edu)

**SI Text**

**Development of PEDOT:PSS-PU-CNT (PPC) Electrode ink**
A 7.5 w/v% poly(3,4-ethylenedioxythiophene):polystyrene sulfonate (PEDOT:PSS) dispersion was prepared in a co-solvent system of deionized (DI) water and dimethyl sulfoxide (DMSO) (85:15 v/v). A 10 w/w% polyurethane (PU) solution was prepared in an ethanol/DI water mixture (95:5 v/v). The PEDOT:PSS and PU precursor solutions were combined at a volume ratio of 1:1.8 (PEDOT:PSS:PU). Homogenization was performed using a dual-syringe mechanical mixing method until a macroscopically uniform dispersion was obtained. Multi-walled carbon nanotubes (CNTs) were subsequently incorporated at 3 wt% relative to the total composite mass. The mixture was further homogenized via repeated syringe-to-syringe extrusion to promote uniform nanofiller distribution within the polymer matrix. Prior to printing, the composite ink was filtered through a 10 µm syringe filter to remove residual aggregates and ensure consistent extrusion through micron-sized nozzles.

**Development of PPC Electrode**

The PDMS encapsulation ink was prepared by mixing Sylgard 186 elastomer base (Part A) and curing agent (Part B) at a 10:1 weight ratio. The mixture was homogenized using a centrifugal mixer (AR-100, Thinky) to ensure uniform dispersion and removal of trapped air bubbles.

The electrode and encapsulation geometries were designed using computer-aided design (CAD) software and exported in DXF format. The DXF files were imported into the printer control software (Nordson EFD), where they were converted into motion commands to control the x–y–z translation of the printing head.

Glass substrates treated with Rain-X water repellent were used as temporary fabrication platforms to facilitate device release after curing. PDMS encapsulation layers were printed using 5 mL syringe barrels fitted with 100 µm inner-diameter nozzles. The PDMS ink was dispensed pneumatically and cured at 125 °C for 15 minutes.

The conductive PPC electrode ink (PEDOT:PSS–PU–CNT) was loaded into 5 mL syringe barrels fitted with 150 µm nozzles. The barrels were connected to an UltimusPlus pneumatic dispenser (Nordson EFD) to apply controlled extrusion pressure.

The adhesive layer was printed onto the skin-contact side of the device using a separate 5 mL syringe barrel containing P4:A6 adhesive ink. The adhesive was deposited in a continuous film and patterned geometry aligned with the electrode’s interface part to ensure conformal contact during application.

**Figure S1. Raman Spectra of PU-PAA matrix.**

**Figure S2. FTIR Spectra of PU-PAA matrix.**

**Figure S3. Viscosity as a function of shear rate for PPC electrode ink.**

**Figure S4. Conductivity comparison of PEDOT:PSS:PU ink with PPC electrode ink.** The box represents the full data range (minimum to maximum), with the center line indicating the median and the square marker denoting the mean. Individual data points are overlaid to show experimental replicates.

**Figure S5. Design and printed PPC electrode with top and bottom encapsulation layer and P4:A6 RCA.**

**Figure S6. Adhesion performance of PPC electrode on skin with P4:A6 RCA.**

**Figure S7. EEG Signal recorded with Ag/AgCl electrode with conductive EEG gel in eyes open and eyes closed paradigm.**

**Figure S8. In vitro cytotoxicity evaluation of the reactivatable conductive adhesive (RCA).**
 a) Representative image of the RCA sample extract on the 12 well plate. b) Cytotoxicity assay of HDFs cultured with different concentrations of RCA extract for 24 hours relative to control
group. Data represents means ± SD from three or four independent experiments. (c) Representative microscopic images of HDFs cultured with (right) and without (left) the RCA extract for 24 hours. Scale bar 100 µm.

**Figure S9.** **Morphological and elemental characterization of P4:A6 RCA film.** Representative SEM image (top left) and corresponding EDS elemental maps of carbon (C), oxygen (O), sulfur (S), and nitrogen (N), together with the overlaid elemental distribution image. Sulfur, originating from the PEDOT conductive component, is distributed uniformly throughout the film without evidence of large-scale aggregation or macroscopic phase separation. The homogeneous distribution of C, O, N, and S indicates good compositional uniformity and effective incorporation of PEDOT within the PU–PAA graft interpenetrating network (g-IPN) matrix. Scale bars: 100 μm.

**Figure S10. Time-dependent volatilization of solvent and conductivity recovery of the reactivatable conductive adhesive (RCA) following activation.**

**Figure S11. Rheological characterization of printable ink.** a) Three-interval thixotropy test (3ITT) showing the viscosity response of P2:A8, P3:A7, P4:A6, and P5:A5 under sequential low-shear, high-shear, and recovery conditions, demonstrating shear-induced viscosity reduction and subsequent structural recovery. b) Shear stress as a function of shear rate for the four ink formulations, illustrating their flow behavior under applied shear.
